# Sphingosine-1-phosphate receptor subtype 1 activation in the central nervous system contributes to morphine withdrawal in rodents

**DOI:** 10.1186/s12974-020-01975-2

**Published:** 2020-10-22

**Authors:** Timothy M. Doyle, Mark R. Hutchinson, Kathryn Braden, Kali Janes, Vicky Staikopoulos, Zhoumou Chen, William L. Neumann, Sarah Spiegel, Daniela Salvemini

**Affiliations:** 1 Henry and Amelia Nasrallah Center for Neuroscience, Saint Louis University School of Medicine, 1402 South Grand Blvd, St. Louis, MO 63104 USA; 2 Department of Pharmacology and Physiology, Saint Louis University School of Medicine, 1402 South Grand Blvd, St. Louis, MO 63104 USA; 3 Discipline of Physiology, University of Adelaide, Adelaide, South Australia 5005 Australia; 4 Institute for Photonics and Advanced Sensing, University of Adelaide, Adelaide, South Australia 5005 Australia; 5 ARC Centre of Excellence for Nanoscale BioPhotonics, University of Adelaide, Adelaide, South Australia 5005 Australia; 6 Department of Pharmaceutical Sciences, School of Pharmacy, Southern Illinois University Edwardsville, 200 University Park, Edwardsville, IL 62026 USA; 7 Department of Biochemistry and Molecular Biology, Virginia Commonwealth University, School of Medicine, 1101 E Marshall St, Richmond, VA 23298 USA

**Keywords:** Sphingosine-1-phosphate, Sphingosine-1-phosphate receptor subtype 1, Naloxone-precipitated withdrawal, GFAP, CD11b, IL-1β

## Abstract

Opioid therapies for chronic pain are undermined by many adverse side effects that reduce their efficacy and lead to dependence, abuse, reduced quality of life, and even death. We have recently reported that sphingosine-1-phosphate (S1P) 1 receptor (S1PR1) antagonists block the development of morphine-induced hyperalgesia and analgesic tolerance. However, the impact of S1PR1 antagonists on other undesirable side effects of opioids, such as opioid-induced dependence, remains unknown. Here, we demonstrate that naloxone-precipitated morphine withdrawal in mice altered de novo sphingolipid metabolism in the dorsal horn of the spinal cord and increased S1P that accompanied the manifestation of several withdrawal behaviors. Blocking de novo sphingolipid metabolism with intrathecal administration of myriocin, an inhibitor of serine palmitoyltransferase, blocked naloxone-precipitated withdrawal. Noteworthy, we found that competitive (NIBR-15) and functional (FTY720) S1PR1 antagonists attenuated withdrawal behaviors in mice. Mechanistically, at the level of the spinal cord, naloxone-precipitated withdrawal was associated with increased glial activity and formation of the potent inflammatory/neuroexcitatory cytokine interleukin-1β (IL-1β); these events were attenuated by S1PR1 antagonists. These results provide the first molecular insight for the role of the S1P/S1PR1 axis during opioid withdrawal. Our data identify S1PR1 antagonists as potential therapeutics to mitigate opioid-induced dependence and support repurposing the S1PR1 functional antagonist FTY720, which is FDA-approved for multiple sclerosis, as an opioid adjunct.

## Introduction

Chronic neuropathic pain is difficult to treat and sufferers are often left with opioids as the only option for some pain relief. However, the long-term use of opioids, such as morphine, is limited by the development of paradoxical painful hypersensitivity (opioid-induced hyperalgesia, OIH) and tolerance to the antinociceptive effects of opioids over time [1, 2]. OIH coupled with tolerance often prompts extended use and escalated dosages that can trigger further changes, which eventually lead to additional unwanted side effects such as dependence, addiction, and abuse [1, 2]. Despite the serious side effects associated with long-term opioid use, this class of drugs remains the gold standard for pain management [3].

**Fig. 1:**
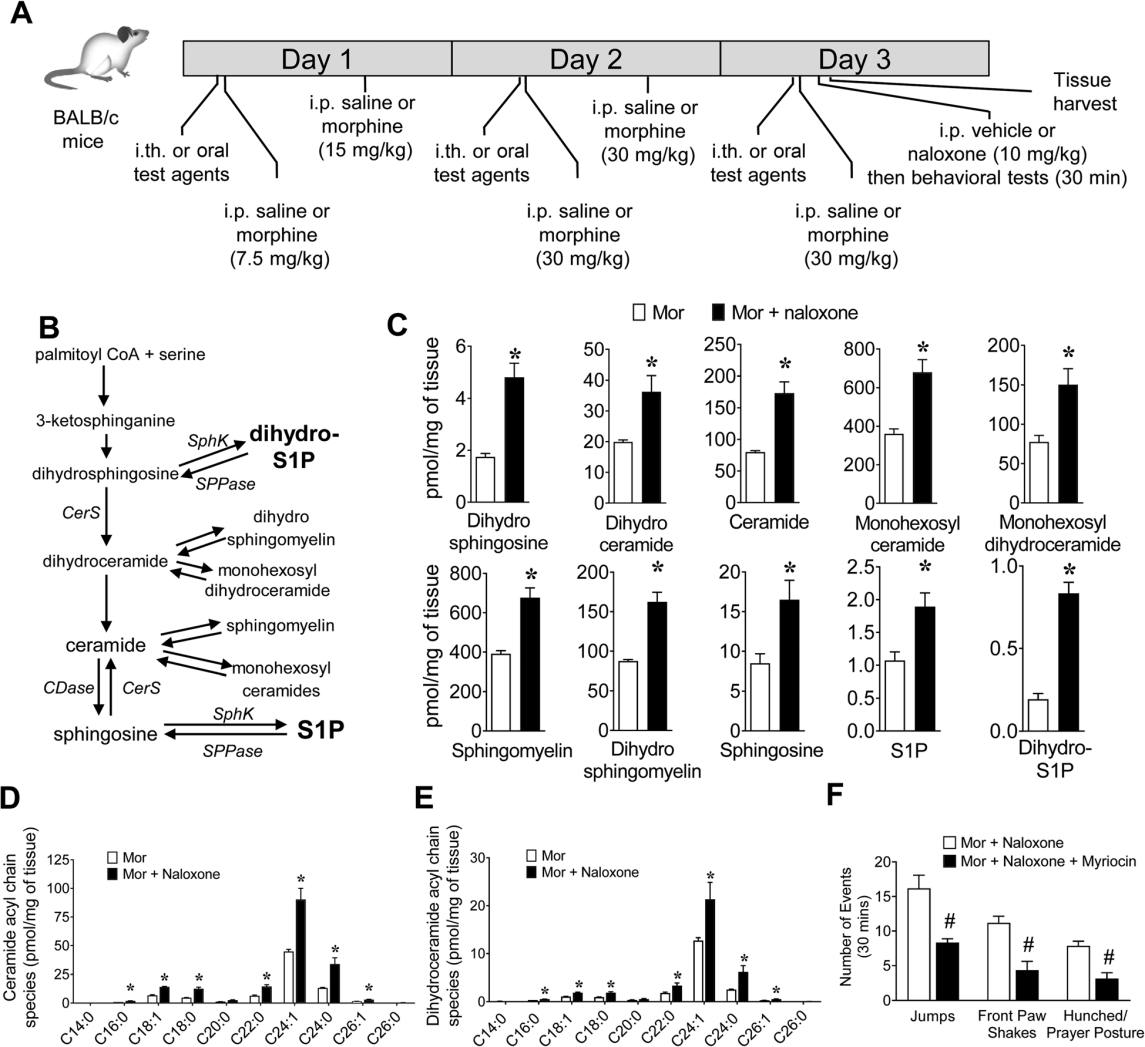
Sphingolipid metabolism is enhanced in the spinal cord during naloxone-precipitated withdrawal and contributes to withdrawal behaviors. (**a**) Schematic of treatment paradigms. (**b**) Schematic of sphingolipid metabolic pathways. (**c-e**) LC-ESI-MS/MS analysis of multiple sphingolipid species of lipids extracted from the mouse spinal cord 1 h after intraperitoneal naloxone (Mor+naloxone, *n* = 5) or its vehicle (Mor; *n* = 6) administration to mice treated with 3 days of escalating morphine treatments (**c**). Naloxone-precipitated withdrawal was associated with significant enhancements of sphingolipid metabolite levels, including S1P and dihydro-S1P, when compared to its vehicle. Individual ceramide (**d**) and dihydroceramide (**e**) species are shown. (**f**) Naloxone-precipitated withdrawal behaviors (e.g., jumping, front paw shaking, and hunched/prayer postures) in mice treated with morphine and naloxone (Mor+naloxone; *n* = 6) were attenuated in mice administered a single daily intrathecal myriocin (300 nM; Mor+naloxone+myriocin; *n* = 6) given 15 min before first dose of morphine of the day. Jumping [*t*(5.8) = 3.9, *p* = 0.008, *d* = 2.28, *n* = 6], front paw shaking [*t*(9.3) = 4.2, *p* = 0.002, *d* = 2.45, *n* = 6], and hunching [*t*(9.9) = 4.4, *p* = 0.0014, *d* = 2.54, *n* = 6]. Data are mean ± SEM and analyzed by two-tailed Welch’s corrected *t* test and Benjamini-Hochberg analysis (**b-d**) or two-tailed Welch’s corrected *t* test (**e**). *q < 0.04 (**b**) or *q* < 0.046 (**c**, **d**) vs. Mor and #*p* < 0.05 vs. Mor

Identifying opioid-sparing approaches that also mitigate the development of dependence, addiction, and abuse requires continued investigation of the molecular underpinnings of how opioids cause such adverse effects. Emerging evidence shows that long-term morphine exposure can lead to dysregulation of sphingolipid metabolism within the dorsal horn spinal cord [4, 5]. Sphingolipids were once thought to serve mainly as structural cellular components, but now are recognized to be potent signaling molecules [6]. Ceramide and sphingosine-1-phosphate (S1P) are among the best-studied sphingolipids [6] and have been implicated in numerous disease states [6, 7], including pain and several of its co-morbidities [8, 9]. S1P is formed from its precursor ceramide produced by activation of enzymes in the sphingomyelin (sphingomyelinase; SMase), and/or de novo (serine palmitoyl transferase, SPT) metabolic pathways (Fig. 1a) [6, 10]. Ceramide is hydrolyzed to sphingosine, which is then phosphorylated by sphingosine kinases to produce S1P [6, 10]. Our recent work uncovered an important link within the central nervous system (CNS) between opioids and sphingolipids in the neurobiology of OIH and antinociceptive tolerance [4, 5]. We found that repeated administration of morphine in rodents altered sphingolipid metabolism in the CNS and increased the levels of ceramide and S1P, which directly contributed to the development of OIH and tolerance [5]. Once formed, S1P is released from cells and initiates autocrine and paracrine signaling by activating any of the five known G protein-coupled S1P receptor subtypes (S1PR1-5) (inside-out signaling) [11]. S1P signaling is terminated by S1P lyase and phosphatases [12]. Except for SIPR4, all S1PRs are found throughout the CNS, but their cell distribution varies [13–16]. We have recently identified that S1PR1 was responsible for transducing the effects of S1P in the development of OIH and tolerance [4]. Its inhibition with S1PR1 functional and competitive antagonists significantly attenuated the development of OIH and tolerance, identifying S1PR1 as a target for therapeutic intervention for OIH and tolerance [4].

The impact of S1PR1 antagonists on other undesirable side-effects of opioids remains unknown. Opioid withdrawal is perhaps one of the most damaging side effects, as the unpleasant physical and affective symptoms associated with withdrawal compel patients to continue taking opioids despite the risks [17]. These symptoms emerge in patients within hours after the last dose and some affective symptoms such as anxiety, depression, and cravings can persist for up to several weeks in chronic users [18]. It is important to find treatments that can help manage these symptoms to facilitate the cessation of opioid use and prevent potential relapse. Guided by our previous work, we now present the first evidence that S1PR1 antagonists may also have a use in opioid-induced dependence.

## Materials and methods

### Study approval

All animal studies were performed in accordance with the International Association for the Study of Pain, the National Institutes of Health guidelines on laboratory animal welfare and approved by the Saint Louis University Institutional Animal Care and Use Committee, and the University of Adelaide Animal Ethics Committee (Ethics approval number M-60-2009).

### Animal

Pathogen-free adult male BALB/c mice (20-30 g starting weight) from Envigo Laboratories (Fredrick, MD, USA) or Laboratory Animal Services (University of Adelaide, Adelaide, AU) were housed 4-5 per cage. All animals were kept in a controlled environment (12 h light/dark cycle) with food and water available ad libitum. Animals were randomly separated into treatment groups for each experiment. Experimenters were blinded to treatments during behavior and biochemical assessments.

### Test compounds

Morphine sulfate was obtained as a kind gift from Mallinckrodt Pharmaceuticals (St. Louis MO, USA) or from the NIDA drug repository (Bethesda MD, USA). Fingolimod (FTY720; Gilenya®) was purchased from Cayman Chemical (Ann Arbor, MI, USA). NIBR-15 was synthesized as previously described [19].

### Experiments

#### Naloxone-precipitated withdrawal

For all studies, chronic morphine dependence was induced as previously described [20] in male BALB/C mice by repeated intraperitoneal (i.p., 0.2 ml) injections of morphine given twice daily (morning and afternoon) for three consecutive days with an escalating dose schedule: day 1 (7.5 and 15 mg/kg), day 2 (30 and 30 mg/kg), and a single dose on day 3 (30 mg/kg). Saline control groups of age-matched male mice received an equal number of saline injections over 3 days. To induce withdrawal behaviors, naloxone (10 mg/kg, i.p., 0.2 ml) was injected 1 h after the last morphine injection on day 3 (Fig. 1a). Animals not receiving naloxone were given an i.p. injection of saline (0.2 ml).

#### Opioid withdrawal behaviors

Behaviors associated with withdrawal in rodents (jumping, front paw shakes, and hunching) [20–23] were measured as previously described [20]. The animals were placed into individual plexiglass observation cylinders (25 cm × 11 cm) and the incidence of jumping, front paw shakes, and hunching were recorded for 30 min.

##### Study 1: Spingolipidomics

Male mice were given the escalating doses of morphine and then treated with saline (*n* = 5) or naloxone (*n* = 6) on day 3. Saline-perfused lumbar portions of the spinal cords were harvested 30 min after behavioral testing for sphingolipidomics.

##### Study 2: Inhibition of morphine withdrawal by myriocin

Male mice were given the escalating doses of morphine (i.p.) in the presence of myriocin (*n* = 6) or vehicle (saline, *n* = 6). Myrioicin (300 nM) or its vehicle was given 15 min before the first morphine injection of morphine each day for 3 days via acute intrathecal (i.th., 5 μL) injection as previously described [24, 25]. The dose of myriocin was chosen from previous studies [5, 26]. All animals received an i.p. injection of naloxone on day 3.

##### Study 3: Inhibition of morphine withdrawal by S1PR1 antagonist

Male mice were given the escalating doses of morphine (i.p.) in the presence of NIBR-15 (3 mg/kg/day; *n* = 4) or vehicle (2-5% DMSO in 0.5% methylcellulose, *n* = 4) or in the presence of FTY720 (0.1 mg/kg/day, *n* = 12) or vehicle (2-5% DMSO in 0.5% methylcellulose, *n* = 11). Test agents were given by oral gavage (0.2 ml) as previously described [27]. The doses of NIBR-15 and FTY720 were chosen from previous studies [27]. All animals received the i.p. injection of naloxone on day 3.

##### Study 4: Neuroinflammation

Male mice were given saline (*n* = 7) or the escalating doses of morphine after oral administration of vehicle (*n* = 6) or FTY720 (0.1 mg/kg/day, *n* = 3). On day 3, saline-treated mice received i.p. saline vehicle injection and all morphine treated mice received the i.p. injection of naloxone. Saline-perfused lumbar portions of the spinal cords were harvested 30 min after behavioral testing for Western blot and ELISA.

### Sphingolipid analysis by mass spectrometry

Sphingolipids were extracted and quantified by liquid chromatography-electrospray ionization-tandem mass spectrometry (LC-ESI-MS/MS) using a 5500 QTRAP (ABI, Ramingham, MA) as previously described [28].

### Western blot analyses

Mouse spinal cords were homogenized in cell lysis buffer containing 1% protease inhibitor cocktail (Sigma-Aldrich, Sydney, Australia, catalog # P8340), sonicated, then centrifuged at 14,000 RPM for 5 min and supernatants collected. Lysate proteins (30 μg determined by BCA assays) were separated on 8% or 10% sodium dodecyl sulfate-polyacrylamide gels and transferred to nitrocellulose. Membranes were treated overnight at 4 °C with antibodies to glial fibrillary acid protein (GFAP) (1:3000, Santa Cruz Biotechnology, Dallas, USA, catalog # sc-6170) or CD11b (1:2000, Santa Cruz Biotechnology, USA, catalog # sc-6614) followed by secondary antibodies for 1.5 h. Immunoblots were incubated for 1 min with the enhanced chemiluminescence detection reagent, and visualized using a LAS 4000 imaging system (GE Healthcare, UK). The absorbance of protein bands of interest were then quantified using the ImageQuant TL software (GE Healthcare, UK). Subsequently, membranes were washed and then immunoblotted with β-actin antibody (1:10,000, Sigma-Aldrich, Australia, catalog # A3854) as a marker of total protein loaded per each lane. GFAP and CD11b protein levels were normalized relative to β-actin levels. Adjustments to blot images for publication were limited to linear brightness and contrast or color inversion using Image J v.1.47 [29] where noted. All blot images were cropped for the clarity of data presentation.

### Cytokine ELISA

The levels of cytokines in spinal cord lysates were assessed using commercially available ELISA kits (R&D Systems, Minneapolis MN, USA) in accordance with the manufacturer’s protocol.

### Statistics

Data are expressed as mean ± SEM for *N* animals as noted. Data were excluded only if animals showed signs of illness not related to study manipulation or intervention or if a data point was considered an outlier by two-tailed Grubb’s test (*p* < 0.05). Sphingolipidomic data were analyzed by two-tailed, Welch’s corrected *t* test and adjusted for the false discovery rate determined by Benjamini-Hochberg method (*Q* < 0.05). All other data were analyzed by two-tailed, Welch’s corrected *t* test or one-way ANOVA with Dunnett’s comparisons with significance determined at *P* < 0.05. All data were analyzed using GraphPad Prism (version 8.0.1 for Windows, GraphPad Software, San Diego CA USA, www.graphpad.com).

## Results and discussion

Mice were treated with escalating doses of morphine (i.p.) before receiving a single i.p. dose of naloxone or vehicle (Fig. 1a). LC-ESI-MS/MS analysis of multiple sphingolipid species in the dorsal horn of the spinal cord harvested 1 h after naloxone or vehicle revealed that sphingolipid metabolism was dramatically altered in mice given naloxone after morphine than mice given the vehicle after morphine (Fig. 1). Specifically, intermediates of de novo biosynthesis of sphingolipids, dihydrosphingosine, and dihydroceramide were increased following naloxone, implicating enhanced de novo biosynthesis of sphingolipids in naloxone-precipitated withdrawal (Fig. 1b, c). Ceramides, monohexosylceramides, monohexosyldihydroceramides, sphingomyelins, and dihydrosphingomyelins were also concomitantly increased (Fig. 1c-d). Importantly, the dysregulation of sphingolipid metabolism led to significant increases in the levels of sphingosine and the bioactive sphingolipid metabolites, S1P, and dihydro-S1P (Fig. 1c). These changes accompanied a significant increase in the incidence of several behaviors that have been long-associated with opioid withdrawal in rodent models (e.g., jumping, front paw shaking, and hunched/prayer postures) (Fig. 1f). To further investigate the link between de novo sphingolipid biosynthesis and the development of withdrawal behaviors, we co-treated mice with myriocin to inhibit serine palmitoyltransferase, the first and rate-limiting enzyme of this pathway [30]. The incidences of naloxone-precipitated withdrawal behaviors were significantly reduced in mice receiving a daily intrathecal injection of myriocin 15 min before the first morphine dose of the day than those receiving its vehicle (Fig. 1f). These results suggest that alterations in de novo sphingolipid biosynthesis within the spinal cord are functionally linked to withdrawal behaviors.

**Fig. 2:**
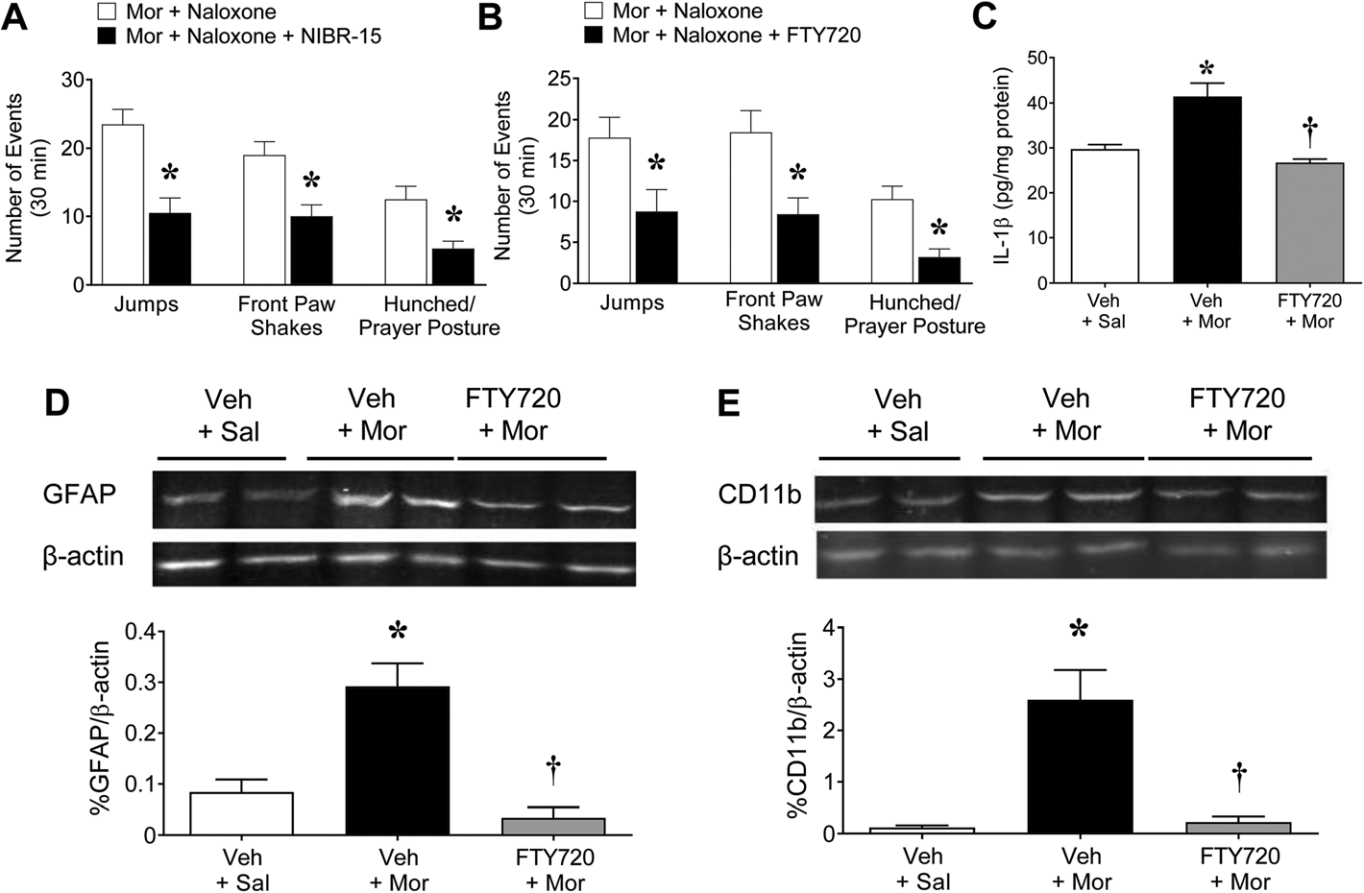
S1PR1 antagonists attenuate naloxone-precipitated withdrawal and associated neuroinflammation in mice. Naloxone-precipitated withdrawal behaviors in mice treated with morphine and naloxone (Mor+naloxone; **a**: *n* = 4, **b**: *n* = 10-11) were attenuated in mice administered oral NIBR-15 (Mor+naloxone +NIBR-15; 3 mg/kg/day; *n =* 4; **a**) or FTY720 (Mor+naloxone+FTY720; 0.1 mg/kg/day; *n =* 12; **b**). Jumping [**a**: *t*(6.0) = 4.1, *p* = 0.0060, *d*_*adj*_ = 1.2; **b**: *t*(20) = 2.5, *p* = 0.023, *d*_*adj*_ = 0.85]; front paw shaking [**a**: *t*(5.9) = 3.5, *p* = 0.014, *d*_*adj*_ = 1.0; **b**: *t*(19) = 3.0, *p* = 0.0070, *d*_*adj*_ = 1.0]; and hunching [**a**: *t*(4.8) = 3.2, *p* = 0.024, *d*_*adj*_ = 0.93; **b**: *t*(17) = 3.8, *p* = 0.0014, *d*_*adj*_ = 1.3]. Naloxone-precipitated withdrawal was associated with increased levels of IL-1β (**c**; *n* = 5), GFAP (**d**; *n* = 6), and CD11b (**e**; *n* = 6) in mice spinal cords (Mor+Nal) compared to mice treated with morphine alone (Mor; **c**: *n =* 7, **d**: *n =* 4, **e**: *n =* 7). Co-administration with FTY720 blocked these events (Mor+Nal + FTY720; 0.1 mg/kg/day; *n* = 3; **c-e**). In **b**, one animal in the Mor+naloxone group was excluded from the jumping data as a high outlier (Grubb’s test). Images have been cropped and adjustments to brightness and contrast were performed across the blots for clarity by Image J [29]. Results are expressed as mean ± SEM and analyzed by (**a**-**b**) two-tailed unpaired Welch’s corrected *t* test. *P < 0.05 vs. Mor+naloxone or (**c**-**e**) two-tailed, one-way ANOVA with Dunnett’s comparisons. [Treatment: (**c**) *F*(2, 13) = 14, *p* = 0.00063, *η*^*2*^ = 0.68; (**d**) *F*(2, 13) = 14, *p* = 0.00060, *η*^*2*^ = 0.68; (**e**) *F*(2, 10) = 9.5, *p* = 0.0049, *η*^*2*^ = 0.65].*p < 0.05 vs. Mor and **†***p* < 0.05 vs. Mor+Nal

Since our sphingolipidomic data revealed that S1P and dihydro-S1P increased in the spinal cord with naloxone-precipitated withdrawal and our previous studies showed the strong contributions of S1PR1 to OIH and tolerance [4], we used the S1PR1 antagonists NIBR-15 and FTY720 to investigate the role of S1PR1 in withdrawal behaviors. NIBR-15 is a potent and highly selective S1PR1 competitive antagonist [19]; whereas, FTY720 is a S1PR1 functional antagonist [31]. FTY720, when phosphorylated by sphingosine kinase 2 to its active counterpart FTY720-P, acts as an S1P agonist at all S1PRs, except S1PR2, but functionally inhibits S1PR1 signaling after binding to S1PR1 and causing sustained depletion of the receptor at the plasma membrane [31]. Mice were given NIBR-15 or FTY720 15 min before the first dose of morphine or saline of the day (Fig. 1a). Co-administration of NIBR-15 or FTY720 (Fig. 2a, b) with morphine significantly reduced the incidence of naloxone-precipitated withdrawal behaviors, unraveling a role for S1PR1.

S1PR1 is found throughout the CNS including the spinal cord and expressed preferentially in glial cells, in particular in astrocytes [13, 16, 32]. Recent evidence suggests that glia play an important role in opioid use disorders [33, 34]. Both astrocytes and microglia within the dorsal horn spinal cord are activated following prolonged exposure to opioids [35–39]. Glial cells are essential for the development of many opioid-induced adverse effects including withdrawal [22, 40, 41].

Once activated, glial cells release various inflammatory cytokines, including tumor necrosis factor (TNF), interleukin-1β (IL-1β), and interleukin-8 (IL-8) [22, 40–42]. During withdrawal, when opioid receptor activation is either spontaneously precipitated or abruptly ceased, this inflammatory environment amplifies neuronal excitability and the withdrawal symptoms [43]. We have recently shown that intrathecal activation of S1PR1 with the highly selective S1PR1 agonist SEW2871 activated the nod-like receptor family, pyrin domain containing 3 (NLRP3) inflammasome and increased IL-1β; these effects occurred predominantly through astrocyte-specific S1PR1 [44]. In mice exhibiting naloxone-precipitated withdrawal behavior, the levels of IL-1β (Fig. 2c) as well as glial markers GFAP (astrocytes; Fig. 2d) and CD11b (microglia/macrophage; Fig. 2d) were significantly increased in the dorsal horn of the spinal cord. These events were significantly attenuated by FTY720 (Fig. 2c-e). This is intriguing in light of the documented role of IL-1β in opioid-induced dependence. Previous studies reveal that morphine-induced IL-1β release in the spinal cord contributes to the development of withdrawal [45–47] and to a broad range of spinal adaptations that contribute to opioid physical dependence [48]. These mechanisms are varied and implicate the ability of IL-1β to enhance presynaptic glutamate release [49] and reduce glial glutamate uptake [50], thus leading to enhanced glutamatergic signaling within the spinal cord [48]. Additionally, IL-1β synergizes with other inflammatory cytokines to enhance interferon-gamma production that can downregulate the potent anti-inflammatory cytokine, interleukin-10 (IL-10) [51, 52]. This imbalance between pro- and anti-inflammatory cytokine productions has been postulated to underlie the increased neuronal excitability in the spinal cord following opioid withdrawal [40, 46]. The mechanisms whereby naloxone-precipitated withdrawal triggers sphingolipid alteration are not known. Previous studies have shown that opioids acting at the μ-opioid receptor can cause receptor internalization and trigger oxidative stress [53] that can enhance central neuroimmune signaling and stimulate sphingolipid metabolism [54]. However, morphine and its metabolite morphine-3-glucudonide can also activate toll-like receptor 4 (TLR4) [55], a pattern recognition receptor that directly activates innate immune and inflammatory pathways. TLR4 is activated following naloxone-precipitated withdrawal [43, 55, 56], and its inhibition ameliorates withdrawal behaviors [57]. TLR4 activation can activate enzymes involved in ceramide and S1P metabolism such as serine palmitoyl-transferase and sphingosine kinase [58, 59]. The role of TLR4 in opioid-induced sphingolipid metabolism dysregulation is under investigation.

Our findings in this study were limited to the effects of S1PR1 signaling had on the physical manifestation of withdrawal in rodent models. These physical symptoms parallel the somatic withdrawal symptoms in humans that include myalgia, hyperalgesia, chills, and stomach cramping [60]. However, opioid withdrawal syndrome also has negative affective aspects that include stress, malaise, emotional pain, anxiety, and depression [60, 61]. Collectively, the physical and affective components provide a negative reinforcement that can motivate opioid seeking behaviors [61]. The affective aspects of opioid withdrawal are quite complex and will require extensive studies to understand how S1PR1 signaling may impact their development. Moreover, our studies focused on the effects in male mice as they have been reported to exhibit greater physical withdrawal symptoms and sensitivity to naloxone (i.e., less naloxone to induce withdrawal) than females [60, 61]. Future studies will need to include the effects in females to parse out any potential sex-dependent difference. Despite these limitations, our current findings remain very exciting because they identify a new area of investigation with a high potential for quick development of desperately needed therapies to treat and prevent opioid withdrawal. Several S1PR1 functional and competitive antagonists have been developed over the last decade. Two S1PR1 functional antagonists are now FDA-approved for the treatment of multiple sclerosis: the pro-drug FTY720 (fingolimod; Gilenya®, Novartis) that was approved in 2010 [31] and ozanimod (RPC1063, Zeposia®, Celgene) that was approved in 2020 [62, 63]. S1PR1 competitive antagonists such as NIBR-15 [19] and TASP0277308 [64] are in advanced preclinical development for a variety of disease states.

## Conclusions

Our findings provide evidence that activation of the de novo pathway and activation of the S1P/S1PR1 axis contributes functionally to morphine-precipitated withdrawal through increased glial cell reactivity and neuroinflammation in male mice. Our findings are promising and warrant further in depth investigation in that they offer a potential strategy whereby FDA-approved S1PR1 antagonists may be used as opioid adjuncts addressing a pressing and unmet medical need.

## Data Availability

All data generated or analyzed during this study are included in this published article.

## Abbreviations

CNS: Central nervous system
GFAP: Glial fibrillary acid protein
IL-1β: Interleukin-1β
IL-8: Interleukin-8
IL-10: Interleukin-10
i.p.: Intraperitoneal
i.th: Intrathecal
LC-ESI-MS/MS: Liquid chromatography-electrospray ionization-tandem mass spectrometry
NLRP3: Nucleotide-binding domain (NOD)-like receptor protein 3
OIH: Opioid-induced hyperalgesia
S1P: Sphingosine-1-phosphate
S1PR1-5: Sphingosine-1-phosphate receptors 1-5
SMase: Sphingomyelinase
SPT: Serine palmitoyl transferase
TLR4: Toll-like receptor 4
TNF: Tumor necrosis factor
